# Democrats and Republicans choose solar panels in very similar ways

**DOI:** 10.3389/fpsyg.2024.1403647

**Published:** 2024-10-16

**Authors:** Nathanael Johnson, Torsten Reimer

**Affiliations:** Brian Lamb School of Communication, Purdue University, West Lafayette, IN, United States

**Keywords:** social identity, solar panels, technology adoption, multi-attribute decision modeling, political identity

## Abstract

**Introduction:**

Appealing to individuals’ social identity is a powerful form of social influence, capable of changing the way people process information, the information they think about, and how they evaluate other individuals. The purpose of this study is to explore the idea that Democrat and Republican environmental norms may impact the attributes and strategies partisans use when choosing whether to have solar panels on a house.

**Methods:**

An online study with *N* = 363 participants was conducted to examine these possible effects through multi-attribute decision making, applying predefined decision process models to participant behavior to test which attribute-based models best describe participants’ decision making. A choice task was combined with an experimental manipulation of political affiliation salience to examine whether the norms of political groups would have influence on decision behavior.

**Results:**

Results of the study show remarkable similarities between political parties in their strategies for choosing solar panels. Members of both political parties appeared to use similar strategies and similar attributes for the formation of their decisions.

**Discussion:**

Recommendations are made that science communicators and policy makers avoid polarizing language so as not to create unnecessary polarization where ideological gaps may not currently exist.

## Introduction

The behavioral sciences have a long history of studying social influence. Behavioral studies have advanced our understanding of social influence through the study of areas like conformity to normative influence (Cialdini and Goldstein, 2004; Reno et al., 1993), credibility (Petty and Cacioppo, 1986; Reimer et al., 2004), and persuasive messaging (Cialdini, 2003; Wolsko et al., 2016). Recently, several researchers have sought to understand social influence by construing it from a decision-making perspective, examining the use of informational cues and attributes and strategies people use to make arguments and choices (Gigerenzer et al., 1999; Reimer et al., 2012; Russell and Reimer, 2018).

We set out to add to this literature by examining how the decision strategies that a decider uses may be impacted by the social identity of the decider. Social identity is known to be a powerful trigger of social influence, changing how people understand the world around them and how they behave. The social identity approach, consisting of Social Identity Theory (Tajfel and Turner, 2004) and Self-Categorization Theory (Turner et al., 1987), assumes that people derive some of their self-concept from group membership (Hogg and Reid, 2006). Salient group memberships can impact what information people choose to look at (Derreumaux et al., 2022; Meffert et al., 2006) and how motivated they are to process information and to be sensitive to the outgroup (van Kleef et al., 2013). As a specific form of social decision making, we suggest that the social identities of deciders may affect the processing of the decision attributes and the strategies that deciders use to make choices.

We test these ideas in the context of solar panel adoption decisions for people who identify as members of either the Democratic or Republican party. These political parties are widely known group memberships that have strong divisions between them (Coggins and Gruschow, 2024; Toprakkiran et al., 2024). Climate change is one of the most known and studied polarizations between these parties. In general terms, people who are more liberal or identify with the Democratic party typically believe more strongly in climate change (Hazboun et al., 2020; Hornsey et al., 2016) and are more concerned about it (Ter-Mkrtchyan et al., 2022) and more willing to take action to mitigate it (Coffey and Joseph, 2013). With the observed differences in environmental beliefs between people who identify with either the Democratic or the Republican party, it might be reasoned that Democrats would also show more interest in technology that is marketed as environmentally friendly, like renewable energy. Many scholars (see for examples Hazboun et al., 2020; Hess et al., 2016; Miniard and Attari, 2021; Yaghoubi et al., 2019) present renewable energy as a significant key to the mitigation of climate change. Furthermore, concern for the environment is both a major driver of renewable energy adoption (Balta-Ozkan et al., 2021; Boudet et al., 2021; Loaiza-Ramírez et al., 2022a) and a central aspect of political identity (Lee et al., 2024). However, this difference in environmental beliefs has not created differences in adoption rates between the parties, with studies showing mixed or nonsignificant results on differences in adoption rates between parties (Crowe, 2020; Dokshin and Gherghina, 2024; Kwan, 2012; Sunter et al., 2018). Furthermore, Mayer and Smith (2024) observed very modest levels of polarization between Democratic and Republican parties in regard to solar tax credits, though the authors anticipate growing polarization in the future. We sought to examine whether there may be differences between the decision processes of members of each of these political parties, even in the absence of differences in overall adoption rates.

We begin by discussing the adoption of solar panels from a partisan perspective, arguing that they may have different reasons for their interest in solar panels. We then formulate specific predictions about what attributes of solar panels might be more important to members of each party. Next, we describe a study that tested these claims. We conclude with a discussion of the results and questions for future research.

### Partisan adoption of solar panels

If environmental concern is often associated with interest in renewable energy, specifically in solar panels, and Democrats are fairly consistently more concerned about the environment than Republicans, then why do we not observe substantial differences in adoption rates? First, it can be observed that solar panels are not just an environmental technology; they are also becoming economically competitive with other energy sources and have the potential to offer a certain level of energy security or backup in the face of a power outage. Scholars have already noted that environmental concerns are neither a sufficient nor a necessary condition to diffuse solar panels through society (Schelly, 2014; Wolske et al., 2017). Renewable energy, although often talked about in conjunction with climate change, is more compatible with conservative ideology than the idea of climate change itself (Hazboun et al., 2020), in that renewable energy can be acceptable for reasons not related to climate change. This is essential to understanding why people who are opposed to the idea of climate change may still take interest in solar panels. Although many scholars and lay people associate renewable energy with climate change mitigation, the adoption of solar panels does not require a concern for the effects of climate change because there are other potential benefits.

Second, there appear to be more differences between political parties in support of renewable energy when it comes to public action, taxes, or mandates, compared to private action. Dupont and Bateman (2012) found in a choice experiment that liberal people are willing to pay more money for goods that benefit the public, such as a utility-scale water softener, compared to conservative people, but the two groups do not differ in their willingness to pay for individually distributed goods that provide private benefits, such as a personal water softener. Similarly, in an environmental context, although members of both parties seem to adopt residential solar panels similarly, support for imposing fees (Miniard and Attari, 2021), taxes (Greenhill et al., 2018), and governmental standards (Hazboun et al., 2020) and spending (Hess et al., 2016) is higher among Democrats and among self-reported liberal people in general (Berkebile-Weinberg et al., 2024).

The differences between Democrats and Republicans in environmental concern and policy support, combined with the lack of difference in solar panel adoption rates, suggest that Democrats and Republicans may be adopting residential renewable energy for different reasons. Therefore, we expected to observe differences between Democrats and Republicans in the information they utilize in choosing whether to have solar panels on a home. Social identity theory posits that the differences between groups will be most significant when group identity is made salient.

### Multi-attribute decision making

To analyze these expected differences, we use multi-attribute decision models (Payne et al., 1988), which describe people’s choices through compensatory and non-compensatory strategies (Gigerenzer et al., 1999; Garcia-Retamero and Dhami, 2009; Reimer and Hoffrage, 2006). Compensatory strategies integrate information on all available choice attributes when making a decision, while non-compensatory strategies stop information search once an attribute is processed that discriminates among choice alternatives (Gigerenzer et al., 1999; Hoffrage and Reimer, 2004; Reimer and Hoffrage, 2006).

Three particular multi-attribute decision models are described in the literature that are utilized in this study, following the three models used in the methodological approach of Garcia-Retamero and Dhami (2009): the take-the-best heuristic (TTB), the unit weight model (UWM), and the weighted additive model (WADD). The TTB is a non-compensatory model that makes decisions based on single attributes in a decision environment (Gigerenzer et al., 1999; Reimer and Hoffrage, 2006). In preference tasks, such as the task described in this study, a TTB begins with the most important attribute of a decision environment and assesses whether the choice alternatives differ in that attribute. If they do, the alternative is chosen that scores more favorably on that attribute. If not, the second-most important attribute is checked, and on down until a discriminating attribute is found. If the choice alternatives have identical values on all given attributes, the model chooses randomly. In this way, the model is always making the decision based on a single criterion, making the model non-compensatory because attributes cannot compensate for one another. The UWM is a compensatory model that uses all available attributes in a decision environment. UWM counts the number of positive attributes of each alternative in the decision environment and chooses the one with the highest overall number of positive attributes, regardless of the relative importance of any attribute. WADD is similar to UWM, in that it uses all attributes in a compensatory way and adds up the number of positive attributes. However, in WADD, each attribute is weighted by how important an individual decider perceives the attribute to be. For both the UWM and WADD, an option is chosen randomly in the case of a tie. For an example of how these processes operate slightly differently in inference tasks, as opposed to preference tasks, see Reimer and Hoffrage (2006). These decision models have been used to describe choice behavior in a variety of areas including airport customs officials choosing passengers to search (Pachur and Marinello, 2013), voting behavior (Graefe and Armstrong, 2011), and predicting home burglary (Garcia-Retamero and Dhami, 2009). Gigerenzer et al. (1999) demonstrated across a variety of scenarios that simple, non-compensatory models such as TTB can be more predictive in certain environments than compensatory and more complex models such as WADD and multiple regression analysis, especially in situations where there is little data. Similarly, research has also described group decision making with these types of models (Reimer and Hoffrage, 2006; Reimer and Katsikopoulos, 2004).

### Attributes and decision models for choosing solar panels

These decision models have not, to our knowledge, been used to study technology adoption of solar panels (Bortoleto et al., 2014). We set out to answer the overall question of what multi-attribute decision strategies describe solar panel adoption decisions and how those strategies may differ across the political aisle. We studied these possible differences in two ways. First, we examined whether Democrats and Republicans differ in the attributes they use to make choices in solar panel adoption and if they assign different levels of importance to attributes. Second, in addition to the possible use of different attributes, members of these two parties may also use different strategies to leverage the attributes to form their decisions. For example, it may be that one party uses fewer attributes than another or that one party’s decision process is best described by single-attribute models. We considered the analysis of the information utilization strategies to be exploratory, as we had no specific expectation as to what differences might be observed. However, the literature on climate change and solar panel adoption, especially in a political context, does suggest several specific ways in which these political parties may differ in their evaluation of relevant attributes of solar panel adoption which informed the attributes that we included in our study and the setup of the study.

#### Money

Money is among the most prevalent and robust variables used to predict technology adoption for those technologies that bring a substantial cost (see Venkatesh et al., 2012). Money itself brings about a state of self-sufficiency, allowing people to feel more independent (Vohs et al., 2006).

The monetary aspect of adopting solar panels is two-sided. On one side, adopting solar panel technology can be expensive, and even though people are often willing to pay more for solar panels or solar energy, compared to fossil fuel energy sources (Kowalska-Pyzalska, 2019; Mamkhezri et al., 2020; Ntanos et al., 2018), the price of purchasing and maintaining solar panels can outweigh people’s willingness to pay (Scarpa and Willis, 2010), though the price of solar panels has dropped significantly over time (Bollinger et al., 2020, 2022). Still, the cost of solar panels is a known concern that impacts residential solar panel adoption (Adepetu et al., 2018), and discounted or subsidized systems tend to sell better (Abdullah Zhou et al., 2017; Gillingham and Bollinger, 2021).

The other side of the monetary consideration is that solar panels can also have a financial return, in that utility bills can be lower, and it is possible to be paid by utility companies for excess electricity generated by the panels (see Aydin et al., 2022; Deng and Newton, 2017), the benefit of which changes based on the price of electricity, solar irradiance (Šúri et al., 2007), and the size and positioning of the panels. The exact production of a solar panel system will vary from house to house, depending on factors like roof size and shape and objects blocking sunlight. Since money is a powerful motivator for people (Vohs et al., 2006) and also a potentially significant barrier to the adoption of solar panels as an upfront cost, it is likely to be a significant attribute for both Republicans and Democrats.

*H1*: Monetary considerations will be an important attribute for both Democrats and Republicans.

#### Environmental friendliness

Often, solar panels are framed as a way to combat climate change, as an alternative energy source from fossil fuels. Solar panel adoption has been robustly associated with both environmental concerns and perceived environmental benefits (Claudy et al., 2013; Lorteau et al., 2024; Palm, 2018; Palm, 2020; Sun et al., 2020), and environmentally minded consumers have a higher willingness to pay for renewable energy, including solar (Danne et al., 2021; Lehmann et al., 2022).

Judging the adoption of solar panels as an environmental behavior, we expect this to be a relevant decision attribute since environmental concern and environmental behaviors are strongly related (Carfora et al., 2017; Judge et al., 2019; Stikvoort and Juslin, 2022). In this way, whether an electricity source is perceived as environmentally friendly is likely to serve as an important decision attribute for those who care about the environment (Loaiza-Ramírez et al., 2022b). Because Democrats tend to be more concerned about the environment (Hornsey et al., 2016), and because Democrats tend to see solar panels more in terms of environmental benefits, compared to Republicans (Horne and Kennedy, 2019), the environmental attribute can be expected to play a larger role for Democrats than for Republicans.

*H2*: The environmental friendliness of an electricity source will be a more important attribute for Democrats than for Republicans.

#### Battery backup

The majority of research on solar panels has focused on solar panels as grid-connected devices without battery backup (Alipour et al., 2020), which is reflective of typical consumers, as a significant majority of residential solar panel systems do not have battery backup (Liang et al., 2022). It is known that individuals who identify as Republicans typically view solar panel systems as a source of self-sufficiency and independence, in comparison to Democrats, who cognitively connect solar panels more strongly with environmental protection (Horne and Kennedy, 2019). Based on this literature, we reasoned that, for Republicans, the presence of a battery may provide comparatively more independence and security in that it allows power during a utility outage (Mayfield, 2019) and may be a particularly important attribute for them. For Democrats, who see solar panels as being about environmental protection, this attribute may be less significant. We therefore predict that this attribute will be weighted more heavily for Republicans than for Democrats.

*H3*: The presence of a battery backup in an electricity source will be a more important attribute for Republicans than for Democrats.

### Political identity salience

Additionally, based on the social identity approach (Tajfel and Turner, 2004), conformity to group norms and prototypes would be expected to increase if a group identity is made salient. Indeed, previous research has observed that framing an environmental topic as a political issue can result in differences between parties that are not present when political affiliations are not made salient (Fielding et al., 2020; Unsworth and Fielding, 2014). Mason (2015) similarly observed that many behaviors have potential to be driven at least partially by partisanship rather than issue importance. We therefore expected that people who have their political identity as a Democrat or Republican made salient will show higher levels of conformity to the stated group norm.

*H4*: The salience of a person’s political party will affect the importance of attributes in the decision making process: Environmental friendliness will be a more important attribute for Democrats in a high salience condition than for Democrats in a low salience condition, and battery backup will be a more important attribute for Republicans in a high salience condition than for Republicans in a low salience condition.

## Methods

Using the online platform Prolific, 400 participants who had indicated in their Prolific profile a general affiliation with either the Democratic or Republican party were recruited to take a survey in exchange for $5. Incomplete responses, responses with failed attention checks (described below), and responses that indicated that people did not identify with either the Democratic or Republican party were removed, leaving a total of 363 usable responses. Of these, 184 self-identified with the Democratic party and 179 self-identified with the Republican party. Participants were an average of 42 years old (*M* = 42.45, *SD* = 13.73) and were 49.6% male (180), 48.2% female (175) and 2.2% self-identified as non-binary/third gender (8). Nearly half of participants (46.8%) reported a Bachelor’s or Associate degree, while 15.1% reported graduate studies, and the remainder indicated no college degree.

### Design

This study was preregistered on the Open Science Framework.^1^ Our analysis does not include the measures of advertisement exposure or one additional measure of attribute perceptions (see below), as these were exploratory measures with no specific hypotheses, and no substantive findings were observed in their regard. A choice task was developed for the study that systematically altered options for utility electricity or solar panel electricity and specific attributes about these options. Three sets of four houses (twelve total, see below) were used as contexts for the choice task. Within each house set, the size of the solar panel (two levels), the presence of a battery (two levels), and a pre-existing level of renewable electricity already at each house (four levels) were systematically combined into 48 total choice alternatives in which each participant would choose whether to accept a solar panel offer for that particular house, with that particular set up (3x2x2x4). Each participant saw all 48 items for a within-subjects design. Additionally, participants were randomly assigned to one of two conditions in a between-subjects design, either high or low political salience. In the high political salience condition, they were asked about their political affiliation and asked to complete a scale about the degree to which they identify with that political group at the beginning of the survey. In the low salience condition, participants did not see these questions until the end of the survey with the general demographic questions. The two conditions were otherwise identical. This political salience manipulation is the only experimental aspect of the design. All other variables, including the actual political affiliation, were not manipulated.

### Procedure

Once participants had agreed to participate, they were introduced to the study and then shown an example. The example explained the information shown to ensure that participants were well-informed on how to make these choices. The participants then were shown the 48 questions regarding whether they would choose solar panels or utility electricity in 48 different scenarios, followed by questions about attributes of the solar panels, the environmental friendliness of solar panels, their exposure to solar panel advertising, and finally demographic questions.

### Materials

Participants were provided with a paragraph that asked them to imagine that they owned a certain house and had received an offer to install solar panels on the house. These paragraphs described the amount of renewable energy that was already at the house from the utility company, whether the solar panel included battery backup, and that the solar panels are rated for 25 years. These paragraphs varied for each of the 48 choice tasks in the renewability and battery information, while the remainder of the paragraph was identical across tasks. Below each paragraph, participants were shown all the available information about the house, solar panel offer, and current electricity setup at the house.

#### General house information

Twelve houses of a median value for their location were chosen from six different cities on January 5th, 2023 from realtor.com. The attributes that were included for each house were the general information from online listings, including a picture of the house, the value of the home, house location, square footage, bedrooms, bathrooms, and the year the house was built. Two houses were chosen from each city, and each city had a paired city in terms of average electricity price in the two cities. Thus, there were three pairs of cities and houses could be grouped in three sets of four for electricity pricing calculations.

#### Utility and solar panel information

Six attributes about the electricity source were provided in each choice set: the wattage of the solar panels, the additional upfront cost, the estimated electricity bill, the amount of home electricity that is from a renewable source, whether there is a battery, and the length of emergency power in case of a power outage.

##### Upfront cost

Panel wattage, production, and pricing are all based on research from the National Renewable Energy Laboratory of the U.S. Department of Energy (Feldman et al., 2020, 2021). We directly used their specific valuations for different system estimates and extrapolated their data to obtain estimates on additional setups.

##### Monthly electricity cost

To estimate the monthly cost of electricity, we used data from the U.S. Energy Information Administration (EIA) and the website Choose Energy,^2^ which provides current prices for electricity by state. We then used the National Renewable Energy Laboratory’s solar power calculator (NREL^3^) to give estimations of solar power generation, based on geographic location and system size.

##### Power outage battery backup

Instead of providing terms like “high resilience” and “low resilience” or specific wattage specifications to participants, we provided a length of time the home could be on emergency power with the solar panel system. These calculations are produced from battery capacity, average energy consumption, and solar panel production.

##### Percentage of renewable electricity

With an annual electricity consumption estimate and an annual solar electricity generation estimate, we also established a baseline amount of renewable electricity from the utility company. Within each of three levels of electricity price, there were four houses with either 0%, a random number between 27 and 33%, a random number between 57 and 63%, or 95% renewable electricity from the utility company. From this, we combined the amount of electricity being supplemented by the solar panels to give a total amount of electricity being renewable with and without solar panels.

### Measures

#### Solar panel choice task

Participants were asked to indicate whether they would accept each solar panel offer at the 48 different houses or to reject the offer and retain the utility setup alone with the question “Would you accept this offer to get solar panels on this home?” with the options of “Do not get solar panels (Keep the utility electricity alone)” and “Accept this offer to add solar panels to the house in addition to the utility electricity” as possible responses.

#### Attribute importance rating

Participants responded to two sets of items, in which the items were identical in the text, but different in the way in which they responded. Participants were informed that these items were not specific to the houses they saw regarding the solar panel choices, instead being general questions about their opinions. For the first set, they were asked “How important are each of the following features for you in deciding whether to put solar panels on a house?” This was rated on a scale of 0 to 10. For the second set, they received the instructions “Please rank order the following in terms of importance in deciding whether to get solar panels on a house. The most important item should be #1, and the least important should be #9.” The items were “When considering getting solar panels, it is important to me to consider (a) whether the panels would come with battery backup; (b) how much money the solar panels would save me on future energy bills; (c) whether the solar panels would be expensive up front; (d) the size of the set of solar panels; (e) whether house is already provided with renewable electricity from the utility company; (f) the square footage of the house; (g) the age of the house; (h) the number of bathrooms in the house, (i) the number of bedrooms in the house.

In addition, participants were provided an open-ended qualitative response question that asked “What else do you consider to be important when it comes to choosing whether to get solar panels?”

#### Environmental perceptions

To measure perceptions of the environmental friendliness of solar panels and battery backups, we included the following items, adapted from Wolske et al. (2017) and rated on a scale of strongly disagree (1) to strongly agree (5). The first three items refer to solar panels alone (*α* = 0.91), while the last three refer to panels with battery backups (α = 0.94). A confirmatory factor analysis showed that the two scales operate as different factors. “Solar panels help slow down climate change,” “If more households get solar panels, environmental quality will improve,” “Having solar panels would be a good way to reduce my environmental impact,” “Solar panels that include a battery backup will help slow down climate change more than solar panels without a battery backup,” “If more households get solar panels with a battery backup, environmental quality will improve more than if people got solar panels without a battery backup,” and “Having solar panels with a battery backup would be a better way to reduce my environmental impact than having solar panels without battery backup.”

#### Political affiliation

To measure political affiliation, participants first responded to a single item asking whether they identify more with the Democratic party, Republican party or neither. To find out how strongly respondents identified with their party, we also administered 12 of the 14 -item group identification scale developed by Leach et al. (2008) and further adapted by Pereira et al. (2021) to the context of political affiliations. Those who self-identified in the single-item measure as a Democrat received items with “Democrat,” with the opposite true for Republicans (α_Democrat_ = 0.95, α_Republican_ = 0.96).

## Results

To examine the results, we compared participant choices against a model that predicts their choices. Our first three hypotheses suggested certain attributes (monetary consideration, environmental friendliness, battery backup) being particularly important in the decision process. To test these hypotheses, we first looked at the attribute rankings and scores to see which attributes participants identified as the most important attributes. Next, we created decision rules to assess how these attributes are being utilized in the decision processes for each group.

### Group identification and overall choice behavior

Democrats (*M* = 3.42, *SD* = 0.79) and Republicans (*M* = 3.49, *SD* = 0.84) reported comparable levels of identification with their own parties [*t*(361) = 0.82, *p* = 0.41]. Participants chose, on average, 25.6 (*SD* = 10.93) solar panels out of a possible 48 (Table 3). Democrats (*M* = 27.17, *SD* = 10.30) chose slightly more solar panels than Republicans (*M* = 24.01, *SD* = 11.35), *t*(361) = 2.78, *p* < 0.01. Democrats also (*M* = 4.11, *SD* = 0.71) perceived solar panels to be more environmentally beneficial than Republicans (*M* = 3.49, *SD* = 1.02), *t*(361) = 6.76, *p* < 0.01. Means, standard deviations, and correlations among several key indexes are presented in Table 1.

**Table 1:** Means, standard deviations, and correlations.

Democrat	*M*	*SD*	1	2	3	4	5	6
Total solar panels	27.17	10.3						
WADD accuracy	0.66	0.12	0.24*					
UWM accuracy	0.64	0.12	0.54*	0.76*				
TTB accuracy	0.64	0.18	0.13	0.28*	0.23*			
Democrat identification	3.4	0.79	0.03	−0.09	−0.03	−0.13		
Solar environmental benefit	4.11	0.71	0.15*	0.07	0.11	0.08	0.33*	
Solar with battery environmental benefit	3.28	0.89	0.11	−0.09	−0.05	−0.04	0.28*	0.28*
Republican	*M*	*SD*	1	2	3	4	5	6
Total solar panels	24.01	11.35						
WADD accuracy	0.64	0.12	0.15*					
UWM accuracy	0.6	0.12	0.54*	0.70*				
TTB accuracy	0.63	0.18	−0.26*	0.12	−0.01			
Republican identification	3.49	0.84	−0.04	−0.12	−0.13	−0.04		
Solar environmental benefit	3.49	1.02	0.30*	−0.03	0.13	−0.14	0.08	
Solar with battery environmental benefit	3.07	1.06	0.24*	−0.14	0.06	−0.08	0.20*	0.68*

This table shows the means, standard deviations, and correlations among the average number of solar panels chosen by each participant, the accuracy of the decision models for each participant, and political identification and perceptions of environmental benefit.

### Decision modeling

In order to model the decisions, we codified the variables as 0 s and 1 s for the models to make predictions of whether the solar panel offer would be accepted or if it would be rejected, retaining the utility setup alone. For the cost of electricity, the difference between the estimated monthly bill with solar panels was subtracted from the estimated bill with only the utility company. Then, a median split was performed on the difference (Median = $110.33). Solar panel options above the median difference between solar panels and utility, were coded as a 1, while solar panel options below the median difference were coded as a 0. Utility options were all coded as a 0.

For batteries, a median split was taken from the houses with batteries in terms of the length of power in an outage, a value of 12.52 h. Solar panel options estimated with more outage time than this were coded as a 1, while options with a lower value than this were coded as a 0, including the utility options and solar panels with no battery backup, which all have an outage length of 0.

For capital costs, all utility setups were coded as a 1, while all solar setups were coded as a 0. The size of solar panels, measured in kilowatts, is coded as a 1 if it is 7.15 kW and a 0 for 3 kW or utility, while the difference in renewable energy is coded as a for solar panels 1 if the panels increase the amount of renewable electricity and a 0 if the percentage of renewable electricity is the same for both utility and solar panel setups. All utility setups were coded as a 0. Finally, given our conceptualization of the panels paying off, we calculated an attribute of paying off, where solar panels receive a 1 if they pay for themselves within 25 years and a 0 if they do not. Utility electricity was coded always as a 1, given that there is no capital cost to pay off.

#### Take-the-best heuristic

A take-the-best heuristic (TTB; see Garcia-Retamero and Dhami, 2009; Gigerenzer et al., 1999; Reimer and Hoffrage, 2006) was modeled using the rank-ordered attributes from participants. For each participant, the attribute they ranked as most important was consulted first, and if it discriminated between the choices, the choice with the higher score on that attribute was selected by the model. If it did not discriminate, the second-ranked attribute was consulted, and down until a discriminating attribute could be used for the model to make a choice. The predicted choices were then compared against the actual choices of participants, and a percentage of correct choices was calculated. Overall, the TTB model predicted 64% of choices accurately, 64% of Democrat choices and 63% of Republican choices, which are not significantly different from one another [*t*(361) = 0.51, *p* = 0.61].

#### Unit weight model

To assess the fit of the unit weight model (UWM; see Gigerenzer et al., 1999), the 1 s and 0 s assigned to each variable (see above) were added together for each of the 48 choice sets (a utility option and a solar panel option for each set) to create a value assigned to each choice. The choice with the greater score was chosen by the model as the predicted outcome. In the case of a tied value, the model chose randomly. This model made identical predictions for every participant, as it ignores participants’ perceived importance of attributes. The UWM was overall 62% accurate in its predictions, being 64% accurate for Democrats and 60% accurate for Republicans, describing Democrat choices better than Republican choices [*t*(361) = 3.63, *p* < 0.01].

#### Weighted additive model

A weighted additive model (WADD; see Garcia-Retamero and Dhami, 2009; Gigerenzer et al., 1999; Reimer and Hoffrage, 2006) was created in a similar way to the UWM, except that the 1 s and 0 s for each variable were multiplied by the perceived importance assigned to each attribute by a participant. Then, the added values were compared against each other within each choice set to make 48 predictions that were unique to each person, since their attribute assessments were also unique to them. Overall, the WADD was 65% accurate, being 66% accurate for Democrats and 64% accurate for Republicans, a difference that was not significant by standard cutoffs but close, [*t*(361) = 1.92, *p = 0*.06].^4^

#### User classification

We further examined the use of these three models by classifying individual participants by how well the models described their behavior. If a model described their choices with at least 75% accuracy, they were classified as a user of this model. TTB described the choices of 102 participants with at least 75% accuracy, while WADD described 93 and UWM described 59 with at least the same accuracy level. These categories overlap somewhat, the three categories accounting for a total of 162 participants, leaving 201 yet unclassified. In the following, we examine how influential the attributes of interest were for those who could be classified by the TTB and WADD models. For those classified as UWM, this is unnecessary, as the model, by definition, assigns all attributes equal weights.

### Money

The first hypothesis states that monetary considerations will be important for both Democrats and Republicans. For both the rankings and ratings of perceived importance, Democrats and Republicans alike indicated that the upfront capital cost, the amount of money saved, and time taken for the solar panels to pay for themselves were three of the top four attributes. Across both groups, the amount of money saved was simultaneously the most important (*M* = 9.05, *SD* = 1.43) and the most highly ranked attribute (*M* = 2.37, *SD* = 1.97). Similarly, the upfront capital costs were the second most important (*M* = 7.91, *SD* = 2.33) and the second most highly ranked (*M* = 2.93, *SD* = 1.78) attribute. And finally, the consideration of the panels paying for themselves within 25 years was in the third place for both importance rating (*M* = 7.42, *SD* = 2.64) and rank ordering (*M* = 3.98, *SD* = 2.09). The only significant differences between the two groups were observed for the upfront capital cost, which were ranked on average slightly higher by Republicans (*M* = 2.74, *SD* = 1.76) than by Democrats (*M* = 3.11, *SD* = 1.78) [*t*(361) = 2.02, *p* = 0.04].^5^

**Figure 1:**
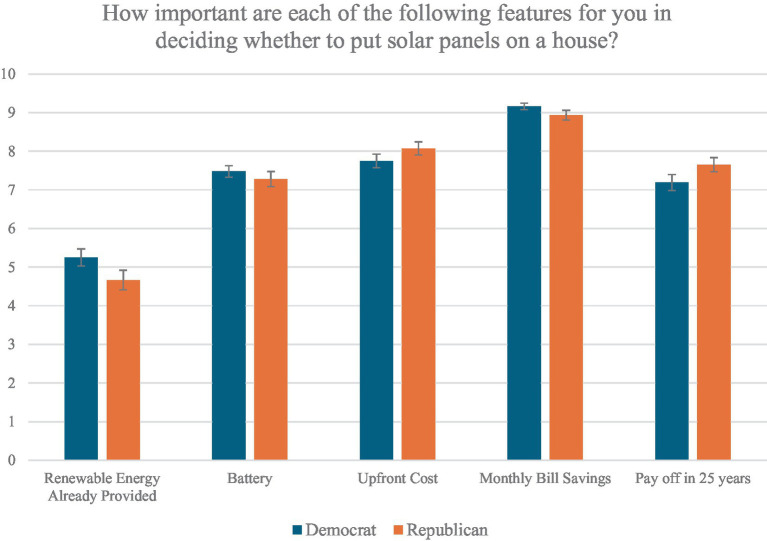
Mean attribute rankings across political parties. This figure shows the mean rating of attributes by Democrats and Republicans when responding to the question “How important are each of the following features for you in deciding whether to put solar panels on a house?”.

Among those who were classified as TTB or WADD deciders, financial considerations were also consistently among the highest ranked attributes. Altogether, 43 Democrats and 39 Republicans, totaling 82 out of 102, had one of the money considerations as their number one attribute and 99 out of 102 had one of these in their top two ranked attributes, indicating that a TTB model very often made predictions based on these attributes. Similarly, among WADD users, monthly bill savings were the most important attribute for both Democrats (*M* = 9.44, *SD* = 1.02) and Republicans alike (*M* = 9.17, *SD* = 1.72), with consideration of whether the panels would pay for themselves in 25 years as second (*M*_Democrats_ = 8.10, *SD*_Democrats_ = 2.41; *M*_Republicans_ = 8.56, *SD*_Republicans_ = 2.03) and the capital cost as third (*M*_Democrats_ = 7.19, *SD*_Democrats_ = 2.61; *M*_Republicans_ = 7.61, *SD*_Republicans_ = 2.03). The WADD strategy, which describes these participants’ choices at least 75% accurately, makes these predictions very heavily based on money, suggesting that these participants do so as well. With these considerations, we consider there to be substantial support for H1, which suggests that the monetary considerations are important for both parties. The means of the attribute rankings for monetary, environmental, and battery considerations are displayed in Figure 1, split by political party.

### Renewable energy

The second hypothesis suggests that the amount of renewable energy at the home will be a more significant attribute for Democrats than for Republicans. In the ranking scale, Democrats (*M* = 5.16, *SD* = 2.00) ranked the amount of renewable energy as more important than Republicans (*M* = 6.01, *SD* = 2.05), *t*(361) = 3.96, *p* < 0.01. In the rating scale of renewable energy, Democrats (*M* = 5.25, *SD* = 2.96) and Republicans did not differ significantly from each other (*M* = 4.66, *SD* = 3.38), [*t*(361) = 1.76, *p* = 0.08]. Put differently, Democrats and Republicans placed the rated renewable electricity similarly, but Democrats assigned it a higher rank-ordered spot on average, showing some indication that Democrats perceived this attribute to be of higher importance than Republicans did.

Among TTB users, only ten Democrats and eight Republicans placed renewable energy in their top three rank-ordered attributes, suggesting that relatively few from either party using this model actually used the renewability of the setup to make their decisions. Among Democrat WADD users, the average weighted score was 5.19 (*SD* = 2.82), while the mean score for comparable Republicans was 3.76 (*SD* = 3.52), which is a significant difference *t*(91) = 2.19, *p* = 0.04. This demonstrates a heavier consideration of this attribute within the prediction models that describe their behavior the best. Furthermore, 43.9% of Republican WADD users scored renewability as either a 0 or 1 (out of 10) in importance, compared to only 11.5% of Democrat WADD users. Combining these findings together, we find first of all that Democrats and Republicans alike seldom made their decisions based solely on the renewability of the setup. However, there is evidence here to show that Democrats did weight renewable energy more heavily than Republicans, in support of H2.

**Figure 2:**
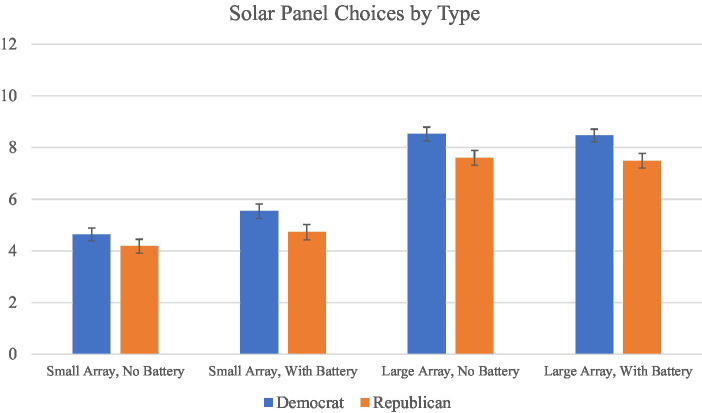
Mean number of times choosing solar panels. This figure shows the mean number of times individual participants chose solar panels when the panel setup **(a)** had a small solar array and no battery, **(b)** had a small solar array and a battery, **(c)** had a large solar array and no battery, and **(d)** had a large solar array and a battery, split by Democrat and Republican respondents.

### Batteries

The third hypothesis suggests that batteries will be a more important decision attribute for Republicans than for Democrats. To test the hypothesis, we first looked at the average ranking and importance assigned to the presence of batteries by participants. In the ranking scale, no significant difference was observed between the two parties, *t*(361) = 0.22, *p* = 0.83. Similarly, in the importance scale, no differences emerged in the average importance ascribed to batteries as a decision attribute, *t*(361) = 0.82, *p* = 0.41. These results show no support for H3.

Additionally, we examined more closely participants who were classified as TTB or WADD users and observed how influential batteries would be in the models predicting their behavior. For both WADD and TTB users, batteries were ranked as the fourth most important attribute by both Democrats and Republicans alike. Furthermore, only seven Democrats and six Republicans among TTB users ranked batteries as the most important attribute, with only nineteen Democrats and sixteen Republicans placing batteries as second or third in importance. Similarly, a mere eight Democrats and eleven Republicans of the 93 WADD users had batteries weighted as a nine or ten, showing that the WADD predicted a high degree of weight for relatively few WADD users. For both of these models, very few participants were predicted to be making their choices based on the presence of a battery, regardless of political party. These models also show no support for H3, and we find altogether no evidence in favor of H3. Furthermore, although participants did rate batteries as more important than attributes like home value or the size of the house when making solar-panel decisions, few participants made decisions based on batteries alone or as one of their top attributes, regardless of political affiliation (Figure 2).

### Political salience

The fourth hypothesis suggested that the conformity to the political norms would depend on the salience of the political identity. No significant differences were found between experimental conditions in the descriptive accuracy of any of the TTB, UWM, or TTB models for either Democrat or Republican participants (all *p*-values >0.06). Similarly, there were no observed differences between conditions for the average rank or average ascribed importance to different attributes. Across all solar-panel attributes, the low- and high-salience conditions were not significantly different from each other within political groups. Additionally, no effects were observed regarding the strength of political identification. Therefore, we find no support for H4.

### Unclassified participants

A total of 201 participants could not be classified by any of the TTB, UWM, or WADD models by a 75% accuracy cutoff point. To begin, we compared the average attribute weights for those who were classified by these models versus those who were not. We found that unclassified participants put heavier weighting ratings on the square footage, age, number of bathrooms, and number of bedrooms, as compared to classified participants (see Table 2), suggesting that these participants may have been looking at contextual variables that may affect the effectiveness of solar panels (such as weather conditions) but are not attributes of solar panels, including features of the house such as the size of a house.

**Table 2:** Attribute weights from classified and unclassified participants.

	Classified	Unclassified	*T*-Test
Battery	6.78	7.87	*t*(361) = 4.57, *p* < 0.001
Monthly bill	9.19	8.94	*t*(361) = 1.70, *p* = 0.090
Capital cost	7.69	8.09	*t*(361) = 1.65, *p* = 0.100
Pays off	7.86	7.06	*t*(361) = 2.88, *p* = 0.004
Panel size	3.44	4.93	*t*(361) = 4.73, *p* < 0.001
Renewability	4.42	5.40	*t*(361) = 2.94, *p* = 0.003
Square feet	3.91	4.79	*t*(361) = 2.60, *p* = 0.010
Home age	3.74	4.67	*t*(361) = 2.85, *p* = 0.005
Bathrooms	1.75	2.78	*t*(361) = 3.57, *p* < 0.001
Bedrooms	2.40	3.45	*t*(361) = 3.11, *p* = 0.002

This table shows the differences in average weighting of attributes between participants who were classified as users of the TTB, UWM, or WADD models and participants who were not classified as using one of these models. Applying a Bonferroni correction to these values with a standard alpha of 0.05 creates a corrected standard of 0.005 for the ten tests. Thus, the difference between groups for Square Feet fails to reach significance with such a correction.

The open-ended response provides some support for this interpretation suggesting that many of the unclassified participants may have indeed been thinking in this way. 50 participants mentioned weather patterns or location as something they considered, either expressing concern about cloudiness and lack of sun exposure or about inclement weather that would either damage the solar panels or make them ineffective. For example, a participant said that when choosing options, they considered “The location of the house in terms of state and my knowledge of the weather/seasons over there.”

This suggests that participants may have been looking at the location of each house to make these choices, rather than the numbers provided as attributes of the panels. An additional nineteen participants expressed that they also took aesthetics and visibility into account when considering the solar panels. A participant stated that they would consider “if it detracts from the aesthetics of the house” to add solar panels. It seems likely, though this wasn’t stated expressly, that these selections may have been made based on the picture of the house. Finally, eleven participants expressed that they looked at the overall value of the home, suggesting that solar panels might not be worthwhile on a less expensive home or wondering whether the solar panels would increase or decrease the value of the home. Additionally, 24 participants expressed that they would want more information on the health or reputation of the installation company or more information about warranties.

**Table 3:** Means, standard deviations, and correlations of solar panel attributes and selections.

	*M*	*SD*	1	2	3	4	5
Total choice	193.69	68.34					
Renewable energy increase	0.35	0.29	0.62*				
Battery duration	7.98	11.16	0.17	−0.08			
Capital cost	23667.71	8519.54	0.26	0.02	0.88*		
Monthly savings	113.34	64.89	0.86*	0.46*	0.21	0.26	
Time to pay off	22.14	12.77	−0.66*	−0.39*	0.32*	0.36*	−0.66*

This table shows how features of the solar panel setup provided in the choice task correlates with the number of times each solar panel setup was chosen among all participants. Renewable energy increase refers to the percentage increase in renewable electricity before installing solar panels to after, battery duration is measured in hours, capital cost and monthly savings are in USD, and time to pay off is measured in years. Features of the setup were necessarily correlated in the design, as they would be realistically significantly correlated in a real purchasing scenario.

## Discussion

We set out to examine whether the previously observed differences between Democrats and Republicans in their concern for the environment would emerge as differences of what information they would take into account when choosing solar panels. We used the information about the homes the panels would be installed on, changes in the amount of renewable electricity, the presence of a battery, and monetary considerations as attributes of study. We did not observe differences between political parties in their assessment of the importance of batteries, but we did observe a difference in the importance of renewability, weighted more for Democrats. However, the influence of both of these attributes was dwarfed by the weight of the monetary considerations, which Democrats and Republicans alike placed as being the most important attributes.

Although there were small differences between the parties, the decision processes that partisans went through to choose solar panels were remarkably similar to each other. These results show far less polarization for residential solar panels than might be inferred from differences in environmental concerns or other political differences. Rather, these results imply that neither Republicans nor Democrats are choosing residential solar panels mainly for environmental or power outage reasons but more commonly for monetary reasons. A growing body of literature has observed that attitudes toward solar panels may not be strongly politically polarized in terms of adoption ties (Crowe, 2020; Dokshin and Gherghina, 2024; Kwan, 2012; Sunter et al., 2018) or even tax credits (Mayer and Smith, 2024), even though qualitative data has suggested that it is polarized (Schelly, 2014). This project contributes to the areas of political and environmental psychology by demonstrating that Democrats and Republicans use similar decision making processes for the adoption of solar panels, indicating that they utilize similar attributes when considering solar panel adoption. Although previous literature might suggest to expect that environmental concerns drive political partisans into different decision making processes, the data presented in this study do not support this expectation, instead suggesting that supporters of both parties utilize similar decision strategies. Although arguing from null results is challenging, with our sample size (*N* = 363), we should have been able to detect even relatively small differences with sufficient power. The observed data do not suggest that there are meaningful differences between these parties in the way that they process solar panel information and make choices.

These results highlight the need, academically and in industry, to explore avenues other than climate change as frames for solar panel adoption, as has been similarly suggested by Wolske et al. (2017), who argued that personal benefits could outweigh perceptions of environmental benefit in solar panel adoption. The presented study moves beyond previous research by demonstrating that the personal benefit drive carries across party lines and by examining the role of personal benefit from a decision modeling perspective. To our knowledge, the methodology of multi-attribute decision modeling has not been used in the study of solar panel adoption. Although solar panel adoption is often framed around climate change in empirical studies, the data presented in this study suggest that it may be that economic and functional frames will be more useful in understanding adoption rates of solar panels in private households. Thus, there may be a mismatch between the reasons that academic scholars are undertaking studies to increase the diffusion rate of solar panels and the reasons that consumers are taking interest. Understanding the information that consumers look at and consider more heavily also has implications for marketing campaigns, in that the highlighting of economic benefits, according to these results, may find more success than highlighting climate benefits, as the monetary considerations seem to carry more weight for most people.

From a political perspective, the findings suggest a lack of polarization in the choices made. However, it has been observed that some of the division between parties in environmental terms emerges most strongly when a situation is made to be political (Fielding et al., 2020; Unsworth and Fielding, 2014), which can lead to inaccurate perceptions of the opposing party (see Geiger et al., 2020). Recent theory development in polarization has argued that an “us” vs. “them” mentality and politically divisive strategies can cause polarization to emerge (McCoy and Somer, 2018), which can grow into a self-reinforcing process if it continues to grow (Axelrod et al., 2021). Polarization therefore may not require actual differences if there are strongly perceived differences. These considerations should encourage science communicators and policy makers to avoid language that suggests an intergroup conflict where in reality, there seems to be no ideological conflict in this specific context.

In our decision modeling and in the open-ended response, we observed a significant diversity of ways that people make their decisions. Although a significant number of people were able to be classified as using the TTB, UWM, or WADD models, there were many that could not be. The open-ended responses suggested that some may not be making their choice based on the actual electricity setup but on the location of the house, concern about how panels will look on the house, or the company that would be installing the panels. These unclassified individuals also put more weight on attributes related to the house itself, such as the number of rooms or the square footage, which suggests a different decision process that we did not capture. Thus, it may well be that a different decision process could be identified that could describe these individuals without involving any of the three major areas of money, battery backup, and environmental attributes that this study focused on.

### Limitations and future directions

One limitation of this study is the lack of an effect of the political salience manipulation. We used a very subtle manipulation by only carrying whether participants were asked about their political identification at the beginning or at the end of the study, but given that there were no effects of the manipulation at all, this was clearly ineffective. Future study could test if stronger manipulation of political salience may be necessary to more robustly speak to a possible effect. It is possible that effects may yet be observed, especially in the environmental attribute, if there were a stronger manipulation. Additionally, although the measure of political affiliation did capture the strength of identification, it did not explicitly measure the expressiveness of a political affiliation. An expressive model of political partisanship sees political affiliation as an enduring and central identity with high levels of emotional attachment (Huddy et al., 2015; Iyengar et al., 2019) and it may be that expressive partisans would show more polarization than those who do not hold political affiliation as an expressive identity. Another limitation refers to the studied sample, which was from an online participant pool and not necessarily representative of the U.S. population, though an approximately even number of Republican and Democrat participants was obtained.

Future studies that seek to follow up on this research should look for other ways in which there might be a Republican “norm,” given that the battery aspect did not appear to pan out as a Republican norm. Future research could also look for differences in decision making strategies from a group salience perspective using either different Democrat-Republican norms of interest, or by examining different groups that may have stronger norms that could be examined. Finally, future research can seek to examine whether a stronger manipulation of political salience or a specific measurement for expressive partisanship would uncover differences not observed here.

## Data Availability

The raw data supporting the conclusions of this article will be made available by the authors, without undue reservation.

## Footnotes

^1^ https://osf.io/s6t5e

^2^ chooseenergy.com

^3^ pvwatts.nrel.gov

^4^For a more conservative estimate with a Bonferroni correction to account for the three t-tests comparing the accuracy of TTB, UWM, and WADD for Republicans and Democrats, a *p*-value of $\frac{.05}{3}=.017$￼ may be used as a cutoff value.

^5^For a more conservative estimate with a Bonferroni correction to account for the six t-tests comparing the differences between Democrats and Republicans on rank ordering and ratings of batteries, renewable energy, and money, a *p*-value of $\frac{.05}{6}=.008$￼ may be used as a cutoff value.
